# Long-Term Visit-To-Visit Blood Pressure Variability and Risk of Diabetes Mellitus in Chinese Population: A Retrospective Population-Based Study

**DOI:** 10.3389/ijph.2023.1605445

**Published:** 2023-02-06

**Authors:** Rui Zhou, Fu-Rong Li, Kuan Liu, Rui-Dian Huang, Hua-Min Liu, Ze-Lin Yuan, Jia-Zhen Zheng, Meng-Chen Zou, Xian-Bo Wu

**Affiliations:** ^1^ Guangdong Provincial Key Laboratory of Tropical Disease Research, Department of Epidemiology, School of Public Health, Southern Medical University, Guangzhou, China; ^2^ School of Medicine, Southern University of Science and Technology, Shenzhen, China; ^3^ Public Health Division, Hospital of Zhongluotan, Guangzhou, China; ^4^ Department of Anaesthesiology, Nanfang Hospital, Southern Medical University, Guangzhou, China; ^5^ Bioscience and Biomedical Engineering Thrust, Systems Hub, The Hong Kong University of Science and Technology (Guangzhou), Guangzhou, China; ^6^ Bioscience and Biomedical Engineering Thrust, Systems Hub, The Hong Kong University of Science and Technology, Hong kong, Hong Kong SAR, China; ^7^ Department of Endocrinology and Metabolism, Nanfang Hospital, Southern Medical University, Guangzhou, China

**Keywords:** China, diabetes mellitus, blood pressure, variability, cohort

## Abstract

**Objectives:** To examine the association between visit-to-visit blood pressure variability (BPV) and incident diabetes mellitus (DM) risk in a Chinese population.

**Methods:** Data comes from China Health and Nutrition Survey (*n* = 15,084). BPV was estimated as the average real variability (ARV) using at least three BP measurements from the year preceding the event and was divided into quartiles. Participants were also categorized into 9 groups on the basis of combinations of systolic BPV (SBPV) and diastolic BPV (DBPV) tertiles. Cox proportional hazards regression models were used.

**Results:** During a median follow-up of 16.8 years, 1,030 (6.8%) participants developed diabetes (incidence rate: 4.65/1,000 person-years). The HRs (95% CIs) for the highest quartile (vs. the lowest quartile) of SBPV and DBPV were 1.60 (1.30–1.97) and 1.37 (1.13–1.67), respectively. Participants with both highest SBPV and DBPV tertile had an ≈89% higher risk of DM (HR, 1.89; 95% CI, 1.47–2.42) compared with those in the both SBPV and DBPV tertile 1 group.

**Conclusion:** Higher SBP ARV and DBP ARV were independently associated with increased risk of incident DM, which was augmented when both presented together.

## Introduction

Diabetes mellitus (DM) is a worldwide pandemic, altering the disease profile around the globe due to a higher incidence of diabetes-specific complications, such as kidney failure and peripheral arterial disease (1). Indeed, DM and its complications have contributed tremendously to the burden of mortality and disability worldwide (2). There were estimated 537 million people with DM worldwide in 2021, and this number is expected to increase to 783 million by 2045 (3). In particular, China has the world’s largest diabetic population, with a prevalence of 12.8% in 2017, which represents more than an estimated 168 million people in China with DM, resulting in a major public health challenge (4). Thus, early detection of patients at high risk for DM is urgently needed.

Elevated blood pressure (BP), which is well known to be associated with cardiovascular disease, is also emerging as a risk factor for DM (5, 6). However, BP fluctuates continually, and these fluctuations tend to remain consistent within patients (7, 8). In the past, BP fluctuations have often been dismissed as random fluctuations and considered to be a limitation of measuring BP in clinical settings (9). In this context, visit-to-visit variability (VVV) of BP, an index that reflects the variability in BP between visits, is being increasingly used as a newer method to evaluate intraindividual BP fluctuations, which has the potential to avoid underestimation of the true risk of elevated BP in the traditional correlation between baseline BP and outcomes of interest, such as cardiovascular disease, mortality, etc (10, 11).

Existing evidence has linked an increased BPV to an increased risk of DM through impaired glucose tolerance following sympathetic overactivity and autonomic imbalance (12). Several observational studies have also demonstrated that high BPV is significantly associated with pre-diabetes and DM (13, 14). However, those studies focused on short-term BPV, and the impact of long-term BPV on DM is less clear. Furthermore, BPV shows differences among different ethnic populations (15), and relevant studies among Asian populations are rare. Although a prior study using data from a nationwide Japanese population demonstrated that visit-to-visit systolic blood pressure variability (SBPV) is higher in subjects with new-onset DM (16), the association of diastolic blood pressure variability (DBPV) on the incidence of DM was less well understood. Therefore, to comprehensively examine the impact of long-term BPV on the risk of DM, the present study aimed to respectively evaluate the association between visit-to-visit SBPV and DBPV and the incidence of DM, as well as the association of different combinations of SBPV/DBPV tertiles on DM risk using a 17-year longitudinal follow-up study of Chinese men and women.

## Methods

The Strengthening the Reporting of Observational Studies in Epidemiology (STROBE) checklist for research reporting of observational studies was followed in this study (Supplementary Table S1).

### Study Population

**FIGURE 1:**
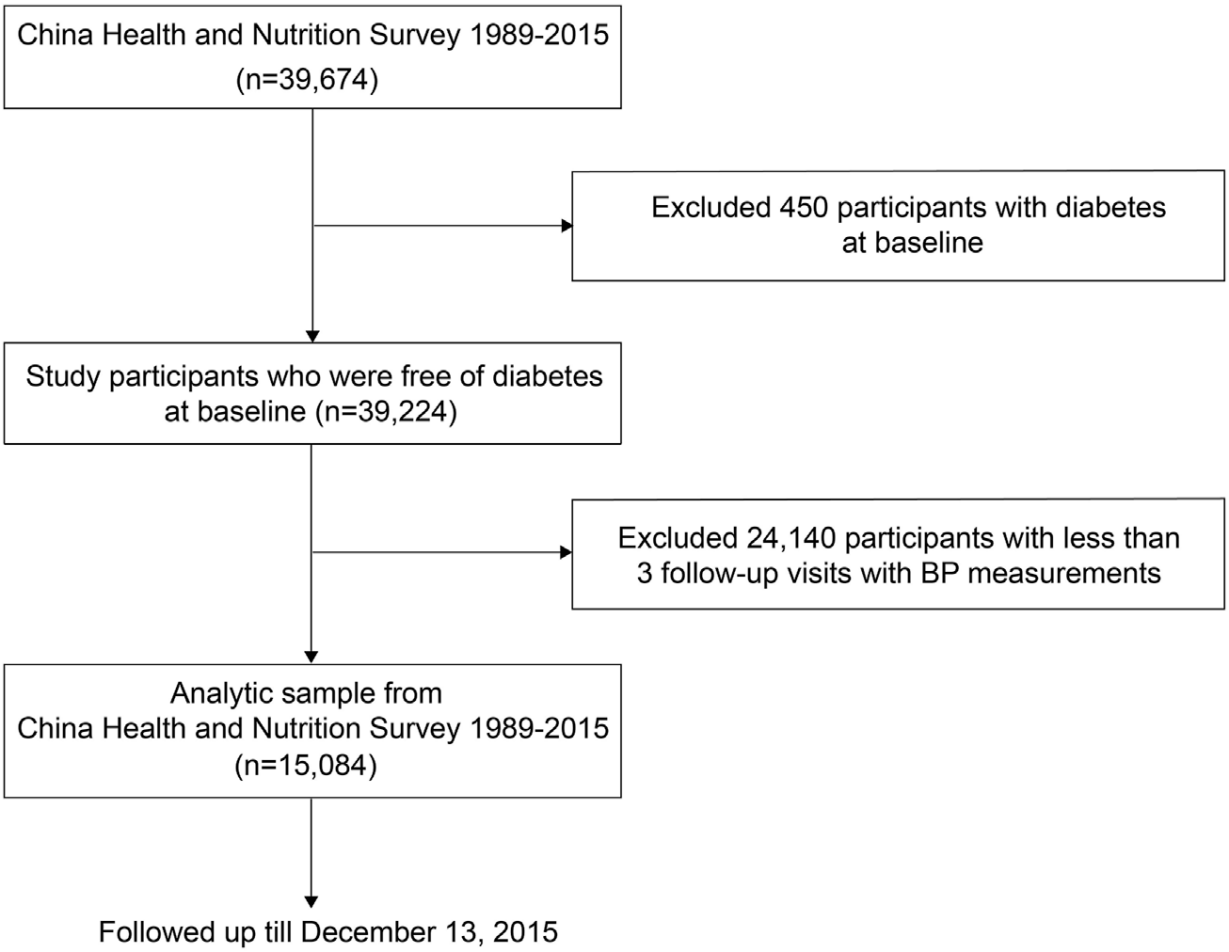
Flowchart of the study population. China Health and Nutrition Survey, China, 1989–2015.

The China Health and Nutrition Survey (CHNS) is an ongoing large-scale, longitudinal, prospective cohort designed to examine the effects of health, nutrition, and family-planning policies and programs in China. Individuals of multiple ages were selected from rural, urban and suburban areas across 15 provinces and municipal cities, using a multistage and random cluster design. A range of demographic, socioeconomic, lifestyle, and health information, including BP, were collected in each wave. The details of the CHNS study have been reported elsewhere (17). This retrospective secondary data analysis was based on 10 waves (from 15 January, 1989 to 13 December 2015) of CHNS survey data. As of 13 December 2015, a total of 39,674 individuals (aged 0–92 years) covering 9,552 households were enrolled. In our study, the following individuals were excluded: participants who had prevalent DM at baseline (*n* = 450) and participants who had fewer than 3 visits with BP measurements (*n* = 24,140). Ultimately, the study sample consisted of 15,084 participants (Figure 1).

The study was approved by the institutional review committees of the Chinese Center for Disease Control and Prevention, and written informed consent was obtained from all participants.

### Baseline BP and Long-Term BPV Measurements

At each wave since 1989, BP was measured according to standard procedure (18), and the average interval of BP measurements across waves was 2.9 years. After at least a 5-min rest, BP was measured by trained health workers or nurses using a standard mercury sphygmomanometer on the right arm in the sitting position with the cuff maintained at heart level. BP was measured three times on one visit, and the three measurements were separated by at least a 30-s interval, during which the right arm was raised up for 5–6 s. BP values measured three times on one visit were averaged and reported as the BP values in this study.

For SBP and DBP, we separately, calculated the long-term mean and BP variability across visits. Based on previous studies (19–22), visit-to-visit BPV was expressed as average real variability (ARV), standard deviation (SD), and coefficient of variation (CV). The BPV formulas were as follows (23): ARV = $\left(\frac{1}{n-1}\right)\sum_{i=1}^{n-1}\left|{BP}_{i+1}-{BP}_{i}\right|$ , SD = $\sqrt{\left(\frac{1}{n-1}\right)\sum_{i=1}^{n}{\left({BP}_{i}-{BP}_{mean}\right)}^{2}}$ , and CV = $\left(\frac{SD}{{BP}_{mean}}\right)*100$ , where n denotes the number of visits with BP measurements, and i is the order of visits. ARV was used for the primary analysis, and CV and SD were used for the secondary analyses. All BPV metrics were analyzed using quartiles of SBPV and DBPV separately (SBPV and DBPV Q1-Q4), and the ARV was also grouped into tertiles to explore the association of different combinations of SBP ARV/DBP ARV tertiles on DM risk.

### Ascertainment of Incident Diabetes

At each follow-up, the participants were asked to report their previous history of diabetes with a questionnaire-based interview based on the following questions: 1) “has a doctor ever told you that you suffer from diabetes? 2) have you received any of the following glucose-lowering treatments, such as a special diet, weight control, oral medications, insulin injections, traditional Chinese medicine, home remedies, or Qi Gong (spiritual method)?” For each visit, participants were diagnosed as DM if at least one of the two answers was yes. In addition, blood samples were collected from patients after overnight fasting by trained nurses and were analyzed at a national central laboratory in Beijing [International Standards Organization (ISO) medical laboratory accreditation certificate 15189:2007] under strict quality control, and the data were available only in 2009. Fasting plasma glucose (FPG) was measured using the glucose oxidase–phenol and 4-aminophenazone (GOD–PAP) method (Randox Laboratories, Ltd., Crumlin, Co., Antrim, United Kingdom) (24). Hemoglobin A1c (HbA1c) was assessed by high-performance liquid chromatography (HLC-723 G7 analyzer; Tosoh Corporation, Tokyo, Japan) (24). Therefore, an additional third criterion (i.e., FPG ≥7.0 mmol/L [126 mg/dL], and/or HbA1c ≥ 6.5% [48 mmol/mol]) (25) was added for outcome ascertainment in 2009. Thus, diabetes diagnosis was ascertained in 2009 if at least one of the two answers concerning diabetes was yes or the third criterion was met.

### Covariates

A standard structured questionnaire was used to collect detailed information on sociodemographic factors, lifestyle factors and medical history. In this study, all baseline covariates with a *P* value less than 0.1 from the univariate analysis were subsequently included in the stepwise logistic regression analysis to identify the most relevant variables contributing to the risk of DN. The result was presented in Supplementary Table S2. Apart from these covariates, several potential cofounders reported to be associated with DM risk were also considered (26–28). Thus, the following covariates were included in our models: age (continuous), sex (men or women), nationality (Han or Minority), region of residence (urban or suburban/rural), education level, smoking status, alcohol consumption, physical activity, BMI (continuous), antihypertensive drugs (yes or no), history of hypertension (yes or no), mean BP (SBP for SBPV and DBP for DBPV). Education level was grouped into three categories: low (graduated from primary school or not educated); medium (graduated from junior middle school or high middle school); high (graduated from college or higher). The highest education level reported in the survey questionnaire was used. Participants were asked about activity levels, which interviewers categorized into five levels (very light, light, moderate, heavy, and very heavy) based on respondents’ descriptions and time spent sitting, standing, walking and lifting heavy loads (29). In our study, we categorized physical activity level as light (very light or light), moderate, or heavy (heavy or very heavy). Smoking status was categorized as non-/ever-smoking and smoking. Alcohol drinking status was categorised as non-drinking and drinking, according to the question “Have you consumed alcohol (beer, wine or other alcoholic beverage) during the past year (yes, no)”? (30). Body mass index (BMI) was measured as weight in kilograms divided by measured height in meters squared. Hypertension was defined by a self-reported diagnosis of hypertension or SBP ≥140 mmHg or DBP ≥90 mmHg based on the 2018 Chinese guidelines for the management of hypertension (31).

### Statistical Methods

Continuous variables are presented as median (25% quartile, 75% quartile), and categorical variables are presented as numbers and percentages. Non-parametric test or Chi-square test were used to assess the difference. The incidence rate of DM was calculated by dividing the number of incident cases by the total follow-up duration (per 1,000 person-years). Cox proportional hazards regression models were used to estimate hazard ratios (HRs) with 95% confidence intervals (CIs) for incident DM. Missing values of all the adjusted covariates accounted for <15.0% and were imputed using the multiple imputation of chained equations (MICEs) method. Characteristics including age, sex, region of residence, and cumulative mean SBP and DBP, were used to impute the missing values. We created 20 imputed data sets and pooled the results using the “mi impute chained” procedure in STATA (version 15; StataCorp, College Station, Texas). The time since the date of the beginning of follow-up was used as the underlying timescale. Participants were followed up from the date they were enrolled in this cohort until the time of incident DM, death, loss to follow-up or were censored at the end of follow-up, whichever occurred first. The proportional hazards assumption was examined using statistical tests and graphical diagnostics based on the scaled Schoenfeld residuals. We divided all measures of BPV into quartiles, using the lowest quartile of BPV as the reference for the regression models. Multivariable adjusted Cox proportional hazards models were applied. Model 1 was adjusted for age, sex, and ethnicity. Model 2 was further adjusted for region of residence, education level, smoking status, alcohol consumption, physical activity level, BMI, and history of hypertension. Model 3 was adjusted for model 2 covariates plus the mean BP (mean SBP for SBPV and mean DBP for DBPV). Tests for linear trend were also performed by entering the median value of each quartile of BPV as a continuous variable in the models. Participants were also divided into 9 groups based on combinations of SBP ARV and DBP ARV tertiles.

We conducted prespecified subgroup analyses within strata of the following factors: age (<50 years or ≥50 years), sex, ethnicity (Han nationality or minority), smoking (yes or no), BMI (<25 kg/m^2^ or ≥25 kg/m^2^), hypertension (yes or no), baseline SBP (<140 mmHg or ≥140 mmHg) and baseline DBP (<90 mmHg or ≥90 mmHg). The results of the subgroup analyses are presented as HRs (95% CIs) of the highest quartile (Q4) compared with the lowest quartile (Q1). The statistical significance of the interactions was assessed by adding a multiplicative term to the Cox models. We also performed several sensitivity analyses to test the robustness of the primary results, including 1) the exclusion of those who received antihypertensive drugs at baseline; and 2) use of the DM definition that only includes self-reports of diabetes diagnosis and/or receipt of any of the glucose-lowering treatment.

Statistical analyses were performed using Stata (version 15; StataCorp, College Station, Texas), and a *P* value < 0.05 was considered to indicate statistical significance.

## Results

### Characteristics of the Study Population

A total of 15,084 participants with at least three waves of BP measurements and without DM at baseline were included in this study. The baseline characteristics of those in the study population who did or did not develop a DM event are presented in Table 1. Overall, the mean age was 31.8 years (SD: 18.4), and women made up 50.5% of the population. The median baseline SBP and DBP was 113.4 mmHg (IQR: 104.0–120.0) and 73.7 mmHg (IQR: 68.7–80.0), respectively. No missing value for the SD, CV, and ARV of BP were found. A comparison of baseline characteristics between included and not included participants is shown in Supplementary Table S3. Supplementary Tables S4, S5 also show the baseline characteristics of these populations categorized by SBP/DBP quartiles. Supplementary Figure S1 indicates that the mean SBP and DBP rose continuously across visits during the follow-up period in both men and women.

**TABLE 1:** Characteristics of participants by incident DM status. China Health and Nutrition Survey, China, 1989–2015.

	Overall (*n* = 15,084)	No DM (*n* = 14,054)	DM (*n* = 1,030)	*P*-value
Baseline variable				
Age, years	32 (19–44)	31 (17–43)	43 (34–53)	<0.001
SBP, mmHg	113.4 (104.0–120.0)	113.4 (102.3–120.0)	120.0 (110.0–130.0)	<0.001
DBP, mmHg	73.7 (68.7–80.0)	73.7 (68.0–80.0)	80.0 (70.0–85.0)	<0.001
BMI, kg/m^2^	21.0 (18.8–23.0)	20.9 (18.7–22.8)	23.2 (21.0–25.8)	<0.001
Hypertension history, %	1,784 (11.8)	1,528 (10.9)	256 (24.9)	<0.001
Antihypertensive drugs, %	1,042 (6.9)	899 (6.4)	143 (13.9)	<0.001
Women, %	7,613 (50.5)	7,078 (50.4)	535 (51.9)	0.328
Ethnicity, %				<0.001
Han	13,092 (86.8)	12,155 (86.5)	937 (91.0)	
Minority	1,950 (12.9)	1,857 (13.2)	93 (9.0)	
Region of residence, %				0.019
Urban	4,621 (30.6)	4,272 (30.4)	349 (33.9)	
Suburban or rural	10,463 (69.4)	9,782 (69.6)	681 (66.1)	
Education level, %				<0.001
Low	8,440 (56.0)	7,816 (55.6)	624 (60.6)	
Medium	5,465 (36.2)	5,106 (36.3)	359 (34.9)	
High	344 (2.3)	318 (2.3)	26 (2.5)	
Physical activity, %				0.084
Light	5,783 (38.3)	5,413 (38.5)	370 (35.9)	
Moderate	4,427 (29.4)	4,131 (29.4)	296 (28.7)	
Heavy	4,656 (30.9)	4,313 (30.7)	343 (33.3)	
Current smoker, %	3,905 (25.9)	3,566 (25.4)	339 (32.9)	<0.001
Current drinker, %	4,497 (29.8)	4,099 (29.2)	398 (38.6)	<0.001
Follow-up variable				
Number of visits with BP measurements	5 (3–10)	5 (3–9)	7 (5–10)	<0.001
BP measurements interval^a^, years	2.9 (2.4–3.7)	2.9 (2.6–3.7)	2.0 (1.7–2.6)	<0.001
SBPV, mmHg				
Mean	115.5 (106.7–125.2)	114.8 (106.1–124.2)	125.0 (116.1–136.3)	<0.001
SD	11.2 (7.8–15.6)	11.1 (7.7–15.4)	12.8 (8.8–17.9)	<0.001
CV	9.8 (6.8–13.3)	9.8 (6.8–13.3)	10.5 (7.3–13.8)	0.002
ARV	11.6 (8.0–16.2)	11.4 (8.0–16.0)	13.4 (9.6–19.1)	<0.001
DBPV, mmHg				
Mean	75.5 (69.8–81.3)	75.1 (69.4–80.8)	80.8 (75.3–86.0)	<0.001
SD	7.8 (5.5–10.4)	7.7 (5.5–10.4)	8.2 (5.8–10.9)	0.001
CV	10.3 (7.4–13.9)	10.3 (7.4–13.9)	10.3 (7.4–13.5)	0.231
ARV	8.3 (57–11.3)	8.2 (5.7–11.3)	8.9 (6.4–11.8)	<0.001

Data are presented as median (25% quartile, 75% quartile) for continuous variables and numbers (percentages) for categorical variables.

Abbreviations: SBP, systolic blood pressure; DBP, diastolic blood pressure; BPV, blood pressure variability; SD, standard deviation; CV, coefficient of variation; ARV, average real variability.

^a^ Follow-up time divided by number of visits.

### Blood Pressure Variability and Risk of DM

Table 2 shows the number and incidence rate of DM events by quartiles of BPV. During a median follow-up of 16.8 years (range: 3.4–26.2), 1,030 (6.8%) participants developed DM (incidence rate: 4.65 per 1,000 person-years). Table 2 also shows the results of the multivariable Cox proportional hazard regressions. A significantly higher risk of DM was observed with higher ARV quartiles in all three adjusted models, although a plateau appeared at the highest DBP ARV quartile in the fully adjusted model (model 3). Specifically, in model 3, SBP ARV quartiles 2 through 4 (compared with the first quartile) were associated with an increased risk of incident DM, with adjusted HRs of 1.27 (95% CI: 1.02–1.57), 1.41 (95% CI: 1.14–1.74), and 1.60 (95% CI: 1.30–1.97), respectively (*P* for trend <0.01). Compared with the lowest DBP ARV quartile, the fully adjusted HRs (95% CIs) of quartiles 2 to 4 were 1.28 (95% CI: 1.05–1.55), 1.37 (95% CI: 1.13–1.67) and 1.37 (95% CI: 1.13–1.67), respectively (*P* for trend = 0.02). When ARV was modeled as a continuous variable, a 1-SD increment in both SBP ARV (HR = 1.11, 95% CI 1.04–1.17) and DBP ARV (HR = 1.09, 95% CI 1.02–1.15) was also significantly associated with an increased risk of DM. Furthermore, a strong, significant trend of increasing DM risk with increasing quartiles of both SBP and DBP variability was observed in Table 2.

**TABLE 2:** HRs and 95% CIs for incident diabetes by BPV quartiles. China Health and Nutrition Survey, China, 1989–2015.

Variable	No. of events/N	Person-years (PYs)	Incidence rate (per 1,000 PYs)	Model 1	Model 2	Model 3
SBP ARV^a^				1.15 (1.09–1.21)	1.15 (1.09–1.22)	1.11 (1.04–1.17)
Quartiles						
Q1	169/3,746	55,024.7	3.07	Ref.	Ref.	Ref.
Q2	228/3,746	64,986.0	3.51	1.30 (1.05–1.60)	1.28 (1.03–1.58)	1.27 (1.02–1.57)
Q3	264/3,744	66,120.0	3.99	1.40 (1.14–1.73)	1.44 (1.17–1.78)	1.41 (1.14–1.74)
Q4	369/3,733	60,634.5	6.09	1.77 (1.45–2.16)	1.74 (1.42–2.13)	1.60 (1.30–1.97)
*P* for trend				<0.01	<0.01	<0.01
**DBP ARV** ^a^				1.12 (1.05–1.19)	1.11 (1.05–1.18)	1.09 (1.02–1.15)
Quartiles						
Q1	195/3,775	55,661.3	3.50	Ref.	Ref.	Ref.
Q2	263/3,731	65,340.7	4.03	1.30 (1.07–1.58)	1.27 (1.05–1.55)	1.28 (1.05–1.55)
Q3	283/3,760	65,148.7	4.34	1.36 (1.12–1.65)	1.38 (1.14–1.68)	1.37 (1.13–1.67)
Q4	289/3,703	60,614.4	4.77	1.45 (1.20–1.76)	1.42 (1.17–1.73)	1.37 (1.13–1.67)
*P* for trend				<0.01	<0.01	0.02

Model 1: adjusted for age, sex, and nationality.

Model 2: adjusted for model 1 plus region of residence, education level, smoking status, alcohol consumption, physical activity, BMI, ever used antihypertensive treatment and history of hypertension.

Model 3: adjusted for model 2 plus mean blood pressure (SBP for SBPV, DBP for DBPV).

Abbreviations: SBPV, SBP variability; DBPV, DBP variability; ARV, the average real variability; HR, hazard ratio; CI, confidence intervals.

^a^ Per SD increment of BPV (measured as ARV).

**FIGURE 2:**
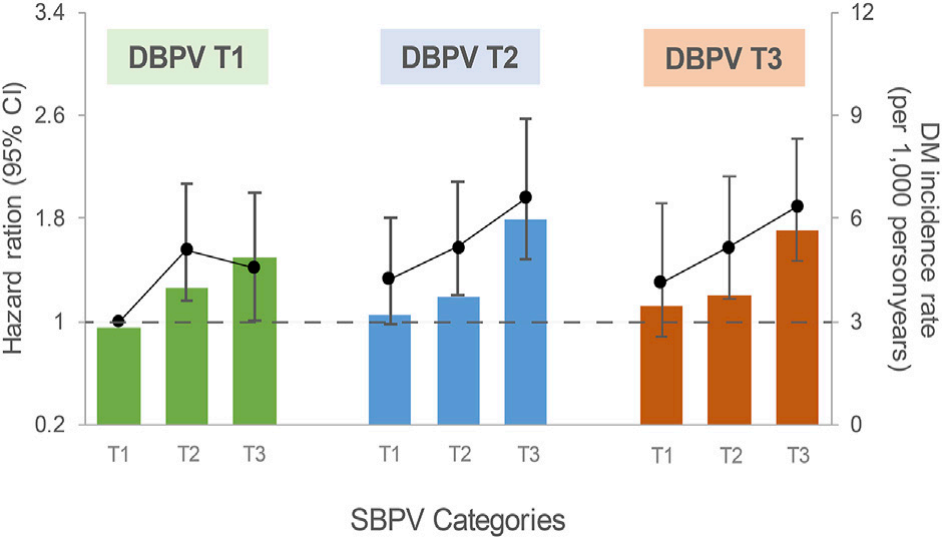
Association between SBPV and incident diabetes by DBPV tertiles. China Health and Nutrition Survey, China, 1989–2015. Models were adjusted for age, sex, nationality, region of residence, education level, smoking status, alcohol consumption, physical activity, BMI, ever used antihypertensive treatment, history of hypertension and cumulative mean BP. The solid black lines represent HRs, and colored columns represent the incidence rate of DM (per 1,000 person-years). Abbreviations: SBPV, systolic blood pressure variability; DBPV, diastolic blood pressure variability; CI, confidence intervals.

We also evaluated the association of different combinations of SBPV/DBPV tertiles with the risk of DM (Figure 2). Compared to participants with both the lowest SBPV and DBPV, those with both the highest SBPV and DBPV tertiles had an elevated risk of DM. The multivariable HRs (95% CIs) of the highest SBPV tertile in each DBP ARV tertile was 1.42 (95% CI: 1.01–2.00), 1.96 (95% CI: 1.49–2.58) and 1.89 (95% CI: 1.47–2.42), respectively, compared with the first SBPV tertile in the first DBP ARV tertile. The highest SBPV tertile in the second DBP ARV tertile showed the highest risk, followed by the third SBPV tertile in the highest DBP ARV tertile.

When alternative variability indexes were used to assess BPV (e.g., SD and CV), the associations were generally diluted and even became null in the fully adjusted models, regardless of the SD or CV (Supplementary Table S6).

### Subgroup Analysis

**FIGURE 3:**
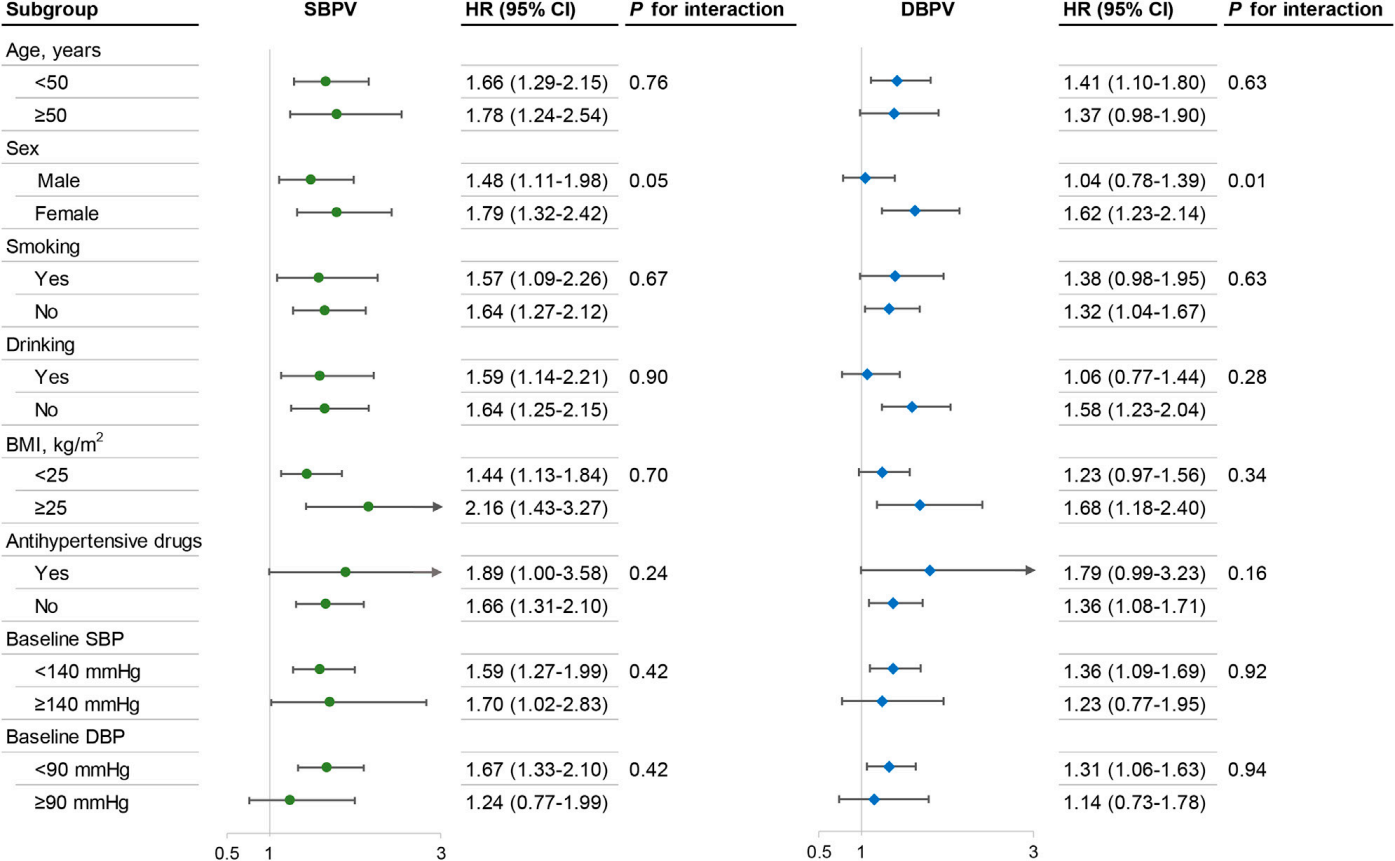
Subgroup analysis of the association between BPV and the risk of diabetes^a^. China Health and Nutrition Survey, China, 1989–2015. Models were adjusted for age, sex, nationality, region of residence, education level, smoking status, alcohol consumption, physical activity, BMI, ever used antihypertensive treatment, history of hypertension, and cumulative mean BP (SBP for SBPV, DBP for DBPV). Abbreviations: SBPV, systolic blood pressure variability; DBPV, diastolic blood pressure variability; BMI, body mass index; HR, hazard ratio; CI, confidence intervals. ^a^The results are presented in terms of the highest *vs.* the lowest quartile of BPV.

Stratified analyses by age, sex, smoking and drinking status, BMI cutoff, hypertension and baseline SBP/DBP cutoffs were conducted (Figure 3). In general, the highest SBPV and DBPV (quartile 4) was significantly associated with a higher risk of DM across various subgroups. However, a higher adjusted HR of DM was observed among women (*P* of 0.05 and 0.01 for SBPV and DBPV interaction, respectively).

Since sex tended to be an effect modifier, we performed additional analyses regarding the sex-specific association of BPV with DM risk (Supplementary Table S7). Overall, the association between BPV and incident DM remained significant with the exception of DBPV among men.

### Sensitivity Analysis

After excluding participants who had received antihypertensive drugs at baseline, the primary results remained largely unchanged (Supplementary Table S8). Likewise, the main results remained robust, though weakened, when the DM definition only included self-reports of a diabetes diagnosis and/or receipt of any glucose-lowering treatment (Supplementary Table S9).

## Discussion

Based on this large-scale, longitudinal study of community-dwelling Chinese men and women, we found that an increased BPV, both SBPV and DBPV, as measured by an increased ARV of intraindividual BP values, was associated with an increased risk of incident DM, independent of baseline characteristics, including hypertension and mean BP. Subgroup analyses revealed that the impact of visit-to-visit BPV on the risk of DM was stronger in women than men.

Our findings strengthen the public health importance of long-term BPV, which might provide additional prognostic information concerning mean BP levels and help to improve risks discrimination for adverse outcomes (7). Until now, few studies have evaluated the association between BPV and incident DM. A previous study based on a large Japanese population demonstrated that a high within-visit BPV was significantly associated with the prevalence of pre-diabetes and DM independent of the mean BP (13). According to other cross-sectional data from the Maastricht study (14), increased 24-h BPV and 7-day home BPV, assessed by the SD of BP, were also linked to an increased risk of type 2 DM. However, those studies merely focused on short-term BPV, and the impact of long-term BPV on DM was less clear. Another prospective study of the Japanese general population reported a positive correlation between long-term SBPV and new-onset DM (16), which is consistent with our findings. Unfortunately, this study did not report the results of DBPV nor the combination of high SBPV and DBPV regarding the risk of DM, and the follow-up period (3 years) was relatively short.

The biological mechanisms underlying the association of long-term BPV with DM remain uncertain. This ambiguity could be partly explained by the contribution of some of the factors that impact long-term BPV to the risk of hyperglycemia, including higher sympathetic nerve activity, lower socioeconomic status, low physical activity, an unhealthy diet, smoking, and sleep apnea (32–36). In addition, a higher long-term BPV may reflect latent metabolic abnormalities (e.g., central obesity and metabolic syndrome [MetS]) and thus relate to DM risk (37, 38). The relationship between long-term BPV and DM can also be explained by BPV as a promoter of endothelial dysfunction and autonomic dysregulation, which may lead to end-organ damage and hyperglycemia (39).

Interestingly, our study also revealed that SBPV had a higher impact than DBPV on DM risk. This result may be partly explained by arterial stiffness, an important factor associated with DM risk, which has been postulated as one of the major causes of SBPV (40). Accumulating evidence in humans and animals has linked arterial stiffness to MetS and its components (e.g., hypertension, hyperglycemia, and obesity), as well as metabolic changes, such as metabolite accumulation, insulin resistance and high free fatty acid concentrations, which may increase the risk of DM (41–44). Furthermore, the highest risk of DM was observed in participants with the highest SBPV tertile and second DBPV tertile, instead of the highest DBPV tertile. The underlying mechanism may be as follows: when DBPV cut-off points exceeded certain limits, components of MetS may not increase consistently but instead tend to reach a plateau (45), leading to a cushion effect of metabolic abnormalities on the risk of DM among those with the highest DBPV (46). Further investigations should highlight the mechanism responsible for the interplay between long-term BPV, MetS, and DM risk.

Another important finding of this study is that the magnitude of the effect of BPV on DM risk was stronger in women than men, regardless of SBPV and DBPV. Similar results were found in a previous observational study (14), which suggested that the associations of 24-h BPV with prediabetes and DM were stronger in women for both day and night. Evidence has shown that BP regulation differs significantly between men and women (47, 48). The autonomic nervous system and its sympathetic arm play important roles in the regulation of BP, and whole-body sympathetic neural activity increases at a greater rate with age in women than in men (49). In addition, the ability to buffer sympathetically mediated vasoconstriction through beta-adrenergic-mediated vasodilation also decreases in women with age (50, 51). Therefore, women have more sympathetic activity than men, resulting in a larger variation in BP. Further research is warranted to examine the sex-specific association between BPV and adverse outcomes.

Compared with the ARV, the SD and CV showed less significance regarding the association of BPV with the risk of DM. Although all three of the above indices are commonly used to assess BPV, only the SD index reflects the dispersion of values around the mean and has been known to be influenced by the average BP level even after adjustment for the average BP level, and neither the SD nor CV account for the order in which BP measurements are obtained (52, 53). Hence, the association between BPV and DM risk might be underestimated when using the SD or CV. In contrast, since the ARV is the average absolute difference between successive BP measurements, which could take into account the sequential order of BP changes while quantifying variability between adjacent readings, it is a more appropriate index of BPV for predicting the risk of DM (54).

Our study adds new evidence that in addition to various adverse outcomes (e.g., cardiovascular events, neurocognitive impairment) (55), BPV measured across visits might be a strong predictor of diabetes, even to a larger extent than average BP values. Given that the use of BPV based on home blood pressure monitoring or ‘in-clinic’ measurements taken over months or years has become increasingly feasible with the proliferation of electronic health records (56), the clinical implication of our study lies in the fact that relatively easily accessible visit-to-visit BPV could improve risk factor stratification for DM, and stabilisation of long-term BP could be considered a potentially important target in reducing risk of DM. BP-lowering therapy, such as calcium-channel blockers or thiazide diuretics (57), may offer benefits in reducing DM risk in high BPV groups.

There were several notable limitations to this study. First, the study was unable to determine causality in the findings because of the observational design. To minimize the possible effects of reverse causality, subjects with preexisting diabetes were excluded, and all subjects with outcomes occurring after the first 3 years of follow-up. Second, the majority of the participants from the original CHNS were not included in this analysis (62%), which might reduce problem of selection bias. Since excluded subjects were more likely to have a history of antihypertensive treatment and a lower educational level (Supplementary Table S3), the association between long-term BPV and risk of DM might have been diluted. Third, although adjustments were made for a wide array of covariates in the Cox regression models, data for several potential confounders (e.g., dietary habits and family history of diabetes) were not available, which might result in some residual confounding. Fourth, BP measurements from each participant across follow-up visits during the entire study duration were not systematically obtained at the same time of day or measured by the same examiner, which could contribute to the random error of BPV and residual confounding, and the associations might be attenuated. Fifth, the diagnosis of DM was mainly based on self-report with only blood samples used in the 2009 survey, leading to relatively higher incident DM cases in the 2009 survey than in other surveys; thus, the Cox model results for time-event estimation may be bias. However, we deemed that this criticism could be assuaged to some extent, given that the primary results remained largely unchanged after performing sensitivity analyses that DM was identified only by self-reports of diabetes diagnosis and/or receiving treatment for diabetes (Supplementary Table S9).

In conclusion, our study adds new evidence that greater long-term variability in both SBP and DBP as assessed by the ARV is independently associated with a significantly increased risk of incident DM after adjusting for baseline characteristics and the cumulative mean BP, and it was augmented when both were present together. Further research is warranted to examine whether interventions for reducing BPV could translate into clinical benefits for the prevention of DM.
