# Supplementary material for: Long-Term Visit-To-Visit Blood Pressure Variability and Risk of Diabetes Mellitus in Chinese Population: A Retrospective Population-Based Study
Source: Int J Public Health. 2023 Feb 6;68:1605445. doi: 10.3389/ijph.2023.1605445 (PMC9939473; doi:10.3389/ijph.2023.1605445)
Supplement: Supplementary file 1 [file DataSheet1.docx]

**Supplementary Files**

**Supplementary Table S1.** STROBE Statement—Checklist of items that should be included in reports of ***cohort studies***. China, 1989-2015

|  | Item No | Recommendation | Page No |
| --- | --- | --- | --- |
| **Title and abstract** | 1 | (*a*) Indicate the study’s design with a commonly used term in the title or the abstract | a, Page 1 |
|  |  | (*b*) Provide in the abstract an informative and balanced summary of what was done and what was found | b, Page 3 |
| Introduction | | | |
| Background/rationale | 2 | Explain the scientific background and rationale for the investigation being reported | Page 4 |
| Objectives | 3 | State specific objectives, including any prespecified hypotheses | Page 5 |
| Methods | | | |
| Study design | 4 | Present key elements of study design early in the paper | Page 5 |
| Setting | 5 | Describe the setting, locations, and relevant dates, including periods of recruitment, exposure, follow-up, and data collection | Page 5 |
| Participants | 6 | (*a*) Give the eligibility criteria, and the sources and methods of selection of participants. Describe methods of follow-up | a, Page 5, 8 |
|  |  | (*b*) For matched studies, give matching criteria and number of exposed and unexposed |  |
| Variables | 7 | Clearly define all outcomes, exposures, predictors, potential confounders, and effect modifiers. Give diagnostic criteria, if applicable | Page 5-9 |
| Data sources/ measurement | 8* | For each variable of interest, give sources of data and details of methods of assessment (measurement). Describe comparability of assessment methods if there is more than one group | Page 5-9 |
| Bias | 9 | Describe any efforts to address potential sources of bias | Page 8-9 |
| Study size | 10 | Explain how the study size was arrived at | Page 5 |
| Quantitative variables | 11 | Explain how quantitative variables were handled in the analyses. If applicable, describe which groupings were chosen and why | Page 5-9 |
| Statistical methods | 12 | (*a*) Describe all statistical methods, including those used to control for confounding | a, Page 7-9 |
|  |  | (*b*) Describe any methods used to examine subgroups and interactions | b, Page 8-9 |
|  |  | (*c*) Explain how missing data were addressed | c, Page 8 |
|  |  | (*d*) If applicable, explain how loss to follow-up was addressed | d, Page 8 |
|  |  | (*e*) Describe any sensitivity analyses | e, Page 9 |
| Results | | |  |
| Participants | 13* | (a) Report numbers of individuals at each stage of study—eg numbers potentially eligible, examined for eligibility, confirmed eligible, included in the study, completing follow-up, and analysed | a, Page 9 |
|  |  | (b) Give reasons for non-participation at each stage | b, Page 5 |
|  |  | (c) Consider use of a flow diagram | c. Figure 1 |
| Descriptive data | 14* | (a) Give characteristics of study participants (eg demographic, clinical, social) and information on exposures and potential confounders | a, Page 9, Table 1. |
|  |  | (b) Indicate number of participants with missing data for each variable of interest | b, na. |
|  |  | (c) Summarise follow-up time (eg, average and total amount) | c, Page 9, Table 2 |
| Outcome data | 15* | Report numbers of outcome events or summary measures over time | Page 9, Table 2 |

| Main results | 16 | (*a*) Give unadjusted estimates and, if applicable, confounder-adjusted estimates and their precision (eg, 95% confidence interval). Make clear which confounders were adjusted for and why they were included | a, Page 9, Table 2, Figure 2. |
| --- | --- | --- | --- |
|  |  | (*b*) Report category boundaries when continuous variables were categorized | b, na. |
|  |  | (*c*) If relevant, consider translating estimates of relative risk into absolute risk for a meaningful time period | c, Page 9, Table 2. |
| Other analyses | 17 | Report other analyses done—eg analyses of subgroups and interactions, and sensitivity analyses | Page 10-11 |
| Discussion | | | |
| Key results | 18 | Summarise key results with reference to study objectives | Page 11 |
| Limitations | 19 | Discuss limitations of the study, taking into account sources of potential bias or imprecision. Discuss both direction and magnitude of any potential bias | Page 13-14 |
| Interpretation | 20 | Give a cautious overall interpretation of results considering objectives, limitations, multiplicity of analyses, results from similar studies, and other relevant evidence | Page 11-14 |
| Generalisability | 21 | Discuss the generalisability (external validity) of the study results | Page 13-14 |
| Other information | | | |
| Funding | 22 | Give the source of funding and the role of the funders for the present study and, if applicable, for the original study on which the present article is based | Page 15, Acknowledgements |

*Give information separately for exposed and unexposed groups.

**Note:** An Explanation and Elaboration article discusses each checklist item and gives methodological background and published examples of transparent reporting. The STROBE checklist is best used in conjunction with this article (freely available on the Web sites of PLoS Medicine at http://www.plosmedicine.org/, Annals of Internal Medicine at http://www.annals.org/, and Epidemiology at http://www.epidem.com/). Information on the STROBE Initiative is available at http://www.strobe-statement.org.

**Supplementary Table S2.** Stepwise logistic regression analysis. China, 1989-2015

| Variable | *P* value |
| --- | --- |
| Age | <0.001 |
| Body mass index | <0.001 |
| Urban residence | 0.008 |
| Current smoker | 0.008 |
| Physical activity | 0.002 |
| Hypertension history | 0.002 |
| Antihypertensive drugs | 0.001 |

**Supplementary Table S3.** Characteristics between included and excluded participants. China, 1989-2015

| Variable | | Included (n=15,084) | Excluded (n=24,590) | *P*-value |
| --- | --- | --- | --- | --- |
| Age, years | | 32 (19-44) | 25.0 (10.0-48.0) | <0.001 |
| SBP, mmHg | | 113.4 (104.0-120.0) | 116.7 (105.0-128.7) | <0.001 |
| DBP, mmHg | | 73.7 (68.7-80.0) | 75.7 (69.3-81.3) | <0.001 |
| BMI, kg/m^2^ | | 21.0 (18.8-23.0) | 21.3 (5.3) | 0.015 |
| Hypertension history, % | | 1,784 (11.8) | 7,205 (29.3) | <0.001 |
| Antihypertensive drugs, % | | 1,042 (6.9) | 1,854 (13.63) | <0.001 |
| Women, % | | 7,613 (50.5) | 13,097 (53.3) | <0.001 |
| Ethnicity, % | |  |  | <0.001 |
|  | Han | 13,092 (86.8) | 20,335 (89.3) |  |
|  | Minority | 1,950 (12.9) | 2,444 (10.7) |  |
| Region of residence, % | |  |  | <0.001 |
|  | Urban | 4,621 (30.6) | 11,125 (47.4) |  |
|  | Suburban or rural | 10,463 (69.4) | 12,327 (52.6) |  |
| Education level, % | |  |  | <0.001 |
|  | Low | 8,440 (56.0) | 7,909 (42.3) |  |
|  | Medium | 5,465 (36.2) | 8,892 (47.6) |  |
|  | High | 344 (2.3) | 1,901 (10.2) |  |
| Physical activity, % | |  |  | <0.001 |
|  | Light | 5,783 (38.3) | 5,323 (28.8) |  |
|  | Moderate | 4,427 (29.4) | 2,462 (13.3) |  |
|  | Heavy | 4,656 (30.9) | 2,462 (13.3) |  |
| Current smoker, % | | 3,905 (25.9) | 5,951 (24.2) | 0.071 |
| Current drinker, % | | 4,497 (29.8) | 4,506 (27.1) | 0.043 |

Data are presented as median (25% quartile, 75% quartile) for continous variables and numbers (percentages) for categorical variables.

Abbreviations: SBP, systolic blood pressure; DBP, diastolic blood pressure.

**Supplementary Table S4.** Baseline characteristics of SBPV quartiles. China, 1989-2015

| Variable | | Quartiles of SBP ARV | | | | *P* value |
| --- | --- | --- | --- | --- | --- | --- |
|  |  | Q1 (0-8.0) | Q2 (8.0-11.6) | Q3 (11.6-16.2) | Q4 (16.2-99.2) |  |
| SBP ARV | | 5.9 (4.4-7.1) | 9.9 (9.0-10.7) | 13.6 (12.5-14.9) | 20.6 (18.1-25.0) | <0.001 |
| Age, years | | 28 (18-39) | 30 (19-40) | 32 (18-43) | 40 (22-54) | <0.001 |
| SBP, mmHg | | 113.0 (105.0-120.0) | 110.7 (101.3-120.0) | 112.0 (100.0-120.0) | 113.4 (105.3-130.0) | <0.001 |
| DBP, mmHg | | 73.7 (70.0-80.0) | 73.7 (68.0-80.0) | 73.7 (66.0-80.0) | 73.7 (70.0-81.3) | <0.001 |
| BMI, kg/m^2^ | | 21.0 (18.9-23.0) | 21.0 (18.8-22.8) | 20.8 (18.5-22.8) | 21.0 (19.1-23.6) | <0.001 |
| Hypertension history, % | | 297 (7.9) | 314 (8.3) | 376 (10.0) | 797 (21.2) | <0.001 |
| Antihypertensive drugs, % | | 196 (5.2) | 196 (5.2) | 221 (5.9) | 429 (11.4) | <0.001 |
| Women, % | | 1,838 (48.7) | 1,902 (50.5) | 1,937 (51.3) | 1,936 (51.4) | 0.072 |
| Ethnicity, % | |  |  |  |  | 0.251 |
|  | Han | 3,264 (86.5) | 3,252 (86.3) | 3,282 (87.0) | 3,294 (87.4) |  |
|  | Minority | 493 (13.1) | 511 (13.6) | 483 (12.8) | 463 (12.3) |  |
| Region of residence, % | |  |  |  |  | <0.001 |
|  | Urban | 1,292 (34.2) | 1,031 (27.4) | 1,101 (29.2) | 1,197 (31.8) |  |
|  | Suburban or rural | 2,481 (65.8) | 2,738 (72.7) | 2,673 (70.8) | 2,571 (68.2) |  |
| Education level, % | |  |  |  |  | <0.001 |
|  | Primary or lower | 1,727 (45.8) | 2,040 (54.1) | 2,219 (58.8) | 2,454 (65.1) |  |
|  | High school | 1,712 (45.4) | 1,466 (38.9) | 1,270 (33.7) | 1,017 (27.0) |  |
|  | Graduate or higher | 145 (3.8) | 71 (1.9) | 59 (1.6) | 69 (1.8) |  |
| Physical activity, % | |  |  |  |  | <0.001 |
|  | Light | 1,558 (41.3) | 1,346 (35.7) | 1,366 (36.2) | 1,513 (40.2) |  |
|  | Moderate | 1,151 (30.5) | 1,122 (30.0) | 1,130 (29.9) | 1,024 (27.2) |  |
|  | Heavy | 999 (26.5) | 1,250 (33.2) | 1,228 (32.5) | 1,179 (31.3) |  |
| Current smoker, % | | 994 (26.4) | 947 (25.1) | 980 (26.0) | 984 (26.1) | 0.105 |
| Current drinker, % | | 1,156 (30.6) | 1,121 (29.7) | 1,100 (29.2) | 1,120 (29.7) | 0.351 |

Data are presented as median (25% quartile, 75% quartile) for continous variables and numbers (percentages) for categorical variables.

Abbreviations: SBP, systolic blood pressure; DBP, diastolic blood pressure.

**Supplementary Table S5.** Baseline characteristics of DBPV quartiles. China, 1989-2015

| Variable | | Quartiles of DBP ARV | | | | *P* value |
| --- | --- | --- | --- | --- | --- | --- |
|  |  | Q1 (0-5.7) | Q2 (5.7-8.3) | Q3 (8.3-11.3) | Q4 (11.3-50.7) |  |
| DBP ARV | | 4.2 (3.0-5.0) | 7.0 (6.4-7.6) | 9.7 (9.0-10.4) | 14.2 (12.5-17.0) | <0.001 |
| Age, years | | 31 (18-42) | 32 (22-43) | 32 (19-44) | 33 (15-48) | 0.001 |
| SBP, mmHg | | 113.4 (105.0-120.0) | 117.7 (102.0-120.0) | 113.3 (102.0-120.0) | 113.4 (104.0-121.3) | <0.001 |
| DBP, mmHg | | 73.7 (70.0-80.0) | 73.7 (68.0-80.0) | 73.7 (68.0-80.0) | 73.7 (68.0-81.3) | <0.001 |
| BMI, kg/m^2^ | | 21.0 (18.9-23.0) | 21.0 (19.0-23.1) | 20.9 (18.8-22.9) | 20.9 (18.4-23.0) | 0.001 |
| Hypertension history, % | | 323 (8.5) | 387 (10.3) | 438 (11.5) | 636 (17.0) | <0.001 |
| Antihypertensive drugs, % | | 198 (5.2) | 232 (6.2) | 259 (6.8) | 353 (9.4) | <0.001 |
| Women, % | | 1,941 (51.1) | 1,931 (51.4) | 1,912 (50.4) | 1,829 (48.9) | 0.124 |
| Ethnicity, % | |  |  |  |  | <0.001 |
|  | Han | 3,361 (88.5) | 3,245 (86.4) | 3,255 (85.8) | 3,231 (86.4) |  |
|  | Minority | 421 (11.1) | 505 (13.5) | 533 (14.1) | 491 (13.1) |  |
| Region of residence, % | |  |  |  |  | <0.001 |
|  | Urban | 1,283 (33.8) | 1,130 (30.1) | 1,123 (29.6) | 1,085 (29.0) |  |
|  | Suburban or rural | 2,513 (66.2) | 2,624 (69.9) | 2,671 (70.4) | 2,655 (71.0) |  |
| Education level, % | |  |  |  |  | <0.001 |
|  | Primary or lower | 1,883 (49.6) | 2,072 (55.2) | 2,158 (56.9) | 2,327 (62.2) |  |
|  | High school | 1,569 (41.3) | 1,426 (38.0) | 1,364 (36.0) | 1,106 (29.6) |  |
|  | Graduate or higher | 134 (3.5) | 91 (2.4) | 58 (1.5) | 61 (1.6) |  |
| Physical activity, % | |  |  |  |  | <0.001 |
|  | Light | 1,597 (42.1) | 1,356 (36.1) | 1,406 (37.1) | 1,424 (38.1) |  |
|  | Moderate | 1,115 (29.4) | 1,114 (29.7) | 1,108 (29.2) | 1,090 (29.1) |  |
|  | Heavy | 1,028 (27.1) | 1,238 (33.0) | 1,225 (32.3) | 1,165 (31.2) |  |
| Current smoker, % | | 962 (25.3) | 967 (25.8) | 980 (25.8) | 996 (26.6) |  |
| Current drinker, % | | 1,146 (30.2) | 1,090 (29.0) | 1,128 (29.7) | 1,133 (30.3) | 0.015 |

Data are presented as median (25% quartile, 75% quartile) for continous variables and numbers (percentages) for categorical variables.

Abbreviations: SBP, systolic blood pressure; DBP, diastolic blood pressure; ARV, the average real variability.

**Supplementary Table S6.** HRs and 95% CIs for incident diabetes by quartiles of BPV measurements. China, 1989-2015

| Variable |  | No. of events/N | Person-years (PYs) | Incidence  rate (per 1,000 PYs) | Model 1 | Model 2 | Model 3 |
| --- | --- | --- | --- | --- | --- | --- | --- |
| **SBPV** |  |  |  |  |  |  |  |
| SD | Q1 | 201/3,744 | 50,972.8 | 3.9 | Ref. | Ref. | Ref. |
|  | Q2 | 213/3,748 | 62,477.9 | 3.4 | 0.92 (0.75-1.14) | 0.96 (0.78-1.18) | 0.96 (0.78-1.18) |
|  | Q3 | 259/3,741 | 66,216.9 | 3.9 | 1.07 (0.88-1.30) | 1.12 (0.92-1.36) | 1.08 (0.89-1.32) |
|  | Q4 | 357/3,736 | 67,097.6 | 5.3 | 1.25 (1.04-1.51) | 1.28 (1.06-1.54) | 1.12 (0.92-1.37) |
| CV | Q1 | 231/3,743 | 51,054.6 | 4.5 | Ref. | Ref. | Ref. |
|  | Q2 | 231/3,741 | 62,754.8 | 3.7 | 0.88 (0.72-1.06) | 0.89 (0.74-1.09) | 0.89 (0.73-1.08) |
|  | Q3 | 280/3,751 | 66,559.4 | 4.2 | 1.12 (0.94-1.35) | 1.18 (0.98-1.43) | 1.14 (0.95-1.38) |
|  | Q4 | 288/3,734 | 66,396.4 | 4.3 | 1.08 (0.90-1.30) | 1.18 (0.98-1.43) | 1.08 (0.89-1.31) |
| **DBPV** |  |  |  |  |  |  |  |
| SD | Q1 | 183/3,745 | 52,247.3 | 3.5 | Ref. | Ref. | Ref. |
|  | Q2 | 243/3,750 | 62,881.7 | 3.9 | 1.00 (0.83-1.20) | 1.00 (0.82-1.21) | 1.00 (0.83-1.21) |
|  | Q3 | 299/3,743 | 65,947.9 | 4.5 | 1.03 (0.85-1.24) | 1.05 (0.87-1.27) | 1.04 (0.86-1.26) |
|  | Q4 | 305/3,731 | 65,688.3 | 4.6 | 1.25 (1.04-1.50) | 1.25 (1.04-1.50) | 1.16 (0.96-1.40) |
| CV | Q1 | 202/3,745 | 52,310.6 | 3.9 | Ref. | Ref. | Ref. |
|  | Q2 | 282/3,744 | 63,242.4 | 4.5 | 1.02 (0.85-1.22) | 1.02 (0.85-1.22) | 1.04 (0.86-1.24) |
|  | Q3 | 312/3,735 | 66,142.9 | 4.7 | 1.06 (0.89-1.27) | 1.14 (0.95-1.37) | 1.15 (0.96-1.38) |
|  | Q4 | 234/3,745 | 65,069.3 | 3.6 | 1.07 (0.89-1.29) | 1.17 (0.97-1.42) | 1.15 (0.95-1.40) |

Model 1: adjusted for age, sex, and nationality.

Model 2: adjusted for model 1 plus region of residence, education level, smoking status, alcohol consumption, physical activity, BMI, ever used antihypertensive treatment and history of hypertension.

Model 3: adjusted for model 2 plus mean blood pressure (SBP for SBPV, DBP for DBPV).

Abbreviations: SBPV, systolic blood pressure variability; DBPV, diastolic blood pressure variability; SD, standard deviation; CV, coefficient of variation; ARV, the average real variability.

**Supplementary Table S7.** HRs and 95% CIs for incident diabetes by quartiles of BPV measurements stratified by sex. China, 1989-2015

| Variable | |  | No. of events/N | Person-years (PYs) | Incidence  rate (per 1,000 PYs) | Model 1 | Model 2 | Model 3 |
| --- | --- | --- | --- | --- | --- | --- | --- | --- |
| **Men** | |  |  |  |  |  |  |  |
|  | SBP ARV | Q1 | 98/1,918 | 30,064.9 | 3.3 | Ref. | Ref. | Ref. |
|  |  | Q2 | 121/1,856 | 33,615.7 | 3.6 | 1.29 (0.97-1.71) | 1.30 (0.98-1.74) | 1.29 (0.97-1.73) |
|  |  | Q3 | 126/1,819 | 33,117.6 | 3.8 | 1.26 (0.95-1.68) | 1.38 (1.03-1.84) | 1.36 (1.01-1.81) |
|  |  | Q4 | 150/1,813 | 30,321.8 | 4.9 | 1.42 (1.08-1.88) | 1.51 (1.14-2.01) | 1.45 (1.08-1.94) |
|  | DBP ARV | Q1 | 104/1,843 | 28,319.2 | 3.7 | Ref. | Ref. | Ref. |
|  |  | Q2 | 129/1,811 | 32,791.1 | 3.9 | 1.19 (0.91-1.56) | 1.22 (0.93-1.60) | 1.21 (0.92-1.59) |
|  |  | Q3 | 137/1,860 | 33,414.5 | 4.1 | 1.18 (0.89-1.55) | 1.22 (0.93-1.61) | 1.21 (0.92-1.60) |
|  |  | Q4 | 125/1,892 | 32,595.2 | 3.8 | 1.10 (0.83-1.45) | 1.14 (0.86-1.50) | 1.09 (0.82-1.45) |
| **Women** | |  |  |  |  |  |  |  |
|  | SBP ARV | Q1 | 71/1,828 | 24,959.9 | 2.8 | Ref. | Ref. | Ref. |
|  |  | Q2 | 107/1,890 | 31,370.3 | 3.4 | 1.33 (0.96-1.83) | 1.28 (0.93-1.76) | 1.26 (0.91-1.74) |
|  |  | Q3 | 138/1,925 | 33,002.4 | 4.2 | 1.59 (1.17-2.16) | 1.58 (1.16-2.14) | 1.52 (1.12-2.07) |
|  |  | Q4 | 219/1,920 | 30,312.7 | 7.2 | 2.15 (1.61-2.88) | 1.94 (1.44-2.60) | 1.74 (1.28-2.36) |
|  | DBP ARV | Q1 | 91/1,932 | 27,342.1 | 3.3 | Ref. | Ref. | Ref. |
|  |  | Q2 | 134/1,920 | 32,549.6 | 4.1 | 1.42 (1.08-1.88) | 1.33 (1.01-1.76) | 1.32 (1.00-1.75) |
|  |  | Q3 | 146/1,900 | 31,734.2 | 4.6 | 1.57 (1.19-2.06) | 1.57 (1.19-2.07) | 1.55 (1.18-2.04) |
|  |  | Q4 | 164/1,811 | 28,019.2 | 5.9 | 1.86 (1.42-2.44) | 1.74 (1.33-2.29) | 1.65 (1.25-2.17) |

Model 1: adjusted for age, sex, and nationality.

Model 2: adjusted for model 1 plus region of residence, education level, smoking status, alcohol consumption, physical activity, BMI, ever used antihypertensive treatment and history of hypertension.

Model 3: adjusted for model 2 plus mean blood pressure (SBP for SBPV, DBP for DBPV).

Abbreviations: SBPV, systolic blood pressure variability; DBPV, diastolic blood pressure variability; ARV, the average real variability.

**Supplementary Table S8.** Sensitivity analyses after excluding 1,042 participants with antihypertensive treatments at baseline (*n*=14,042). China, 1989-2015

| Variable | | No. of events/N | Person-years (PYs) | Incidence  rate (per 1,000 PYs) | Model 1 | Model 2 | Model 3 |
| --- | --- | --- | --- | --- | --- | --- | --- |
| SBP ARV | |  |  |  |  |  |  |
|  | Q1 | 152/3,552 | 52733.7 | 2.9 | Ref. | Ref. | Ref. |
|  | Q2 | 207/3,551 | 62325.4 | 3.3 | 1.29 (1.03-1.61) | 1.27 (1.01-1.59) | 1.26 (1.00-1.58) |
|  | Q3 | 225/3,530 | 63117.6 | 3.6 | 1.34 (1.07-1.67) | 1.39 (1.11-1.73) | 1.35 (1.08-1.69) |
|  | Q4 | 303/3,307 | 55296.5 | 5.5 | 1.73 (1.40-2.15) | 1.73 (1.39-2.15) | 1.60 (1.28-2.00) |
| DBP ARV | |  |  |  |  |  |  |
|  | Q1 | 172/3,577 | 53444.5 | 3.2 | Ref. | Ref. | Ref. |
|  | Q2 | 234/3,501 | 62297.3 | 3.8 | 1.34 (1.09-1.65) | 1.30 (1.06-1.60) | 1.30 (1.05-1.60) |
|  | Q3 | 248/3,504 | 61667.8 | 4.0 | 1.40 (1.13-1.72) | 1.40 (1.14-1.73) | 1.39 (1.13-1.71) |
|  | Q4 | 233/3,358 | 56063.8 | 4.2 | 1.45 (1.18-1.79) | 1.41 (1.14-1.75) | 1.34 (1.09-1.66) |

Model 1: adjusted for age, sex, and nationality.

Model 2: adjusted for model 1 plus region of residence, education level, smoking status, alcohol consumption, physical activity, BMI and history of hypertension.

Model 3: adjusted for model 2 plus mean blood pressure (SBP for SBPV, DBP for DBPV).

Abbreviations: SBPV, systolic blood pressure variability; DBPV, diastolic blood pressure variability; ARV, the average real variability.

**Supplementary Table S9.** Sensitivity analyses when DM was identified only by self-reports of a history of diabetes diagnosis, and/or receiving treatment for diabetes (*n*=15,157, DM cases=604). China, 1989-2015

| Variable | | No. of events/N | Person-years (PYs) | Incidence  rate (per 1,000 PYs) | Model 1 | Model 2 | Model 3 |
| --- | --- | --- | --- | --- | --- | --- | --- |
| SBP ARV | |  |  |  |  |  |  |
|  | Q1 | 142/3,452 | 51,381.5 | 2.8 | Ref. | Ref. | Ref. |
|  | Q2 | 192/3,434 | 60,389.6 | 3.2 | 0.96 (0.73-1.26) | 0.95 (0.67-1.33) | 0.94 (0.67-1.33) |
|  | Q3 | 205/3,377 | 60,523.7 | 3.4 | 1.19 (0.92-1.53) | 1.25 (0.91-1.72) | 1.21 (0.89-1.61) |
|  | Q4 | 235/2,946 | 49,320.6 | 4.8 | 1.44 (1.13-1.84) | 1.57 (1.17-2.12) | 1.50 (1.06-1.89) |
| DBP ARV | |  |  |  |  |  |  |
|  | Q1 | 156/3,453 | 51,733.1 | 3.0 | Ref. | Ref. | Ref. |
|  | Q2 | 207/3,347 | 59,770.2 | 3.5 | 1.10 (1.86-1.42) | 1.08 (0.80-1.46) | 1.08 (0.80-1.46) |
|  | Q3 | 222/3,328 | 58,503.5 | 3.8 | 1.46 (1.15-1.86) | 1.31 (0.98-1.75) | 1.29 (0.97-1.73) |
|  | Q4 | 189/3,081 | 51,608.6 | 3.7 | 1.55 (1.21-1.97) | 1.42 (1.06-1.89) | 1.33 (1.03-1.72) |

Model 1: adjusted for age, sex, and nationality.

Model 2: adjusted for model 1 plus region of residence, education level, smoking status, alcohol consumption, physical activity, BMI, ever used antihypertensive treatment and history of hypertension.

Model 3: adjusted for model 2 plus mean blood pressure (SBP for SBPV, DBP for DBPV).

Abbreviations: SBPV, systolic blood pressure variability; DBPV, diastolic blood pressure variability; ARV, the average real variability.

**Supplementary Figure S1.** The sex-specific mean SBP and DBP across follow-up years for the entire study duration. China, 1989-2015


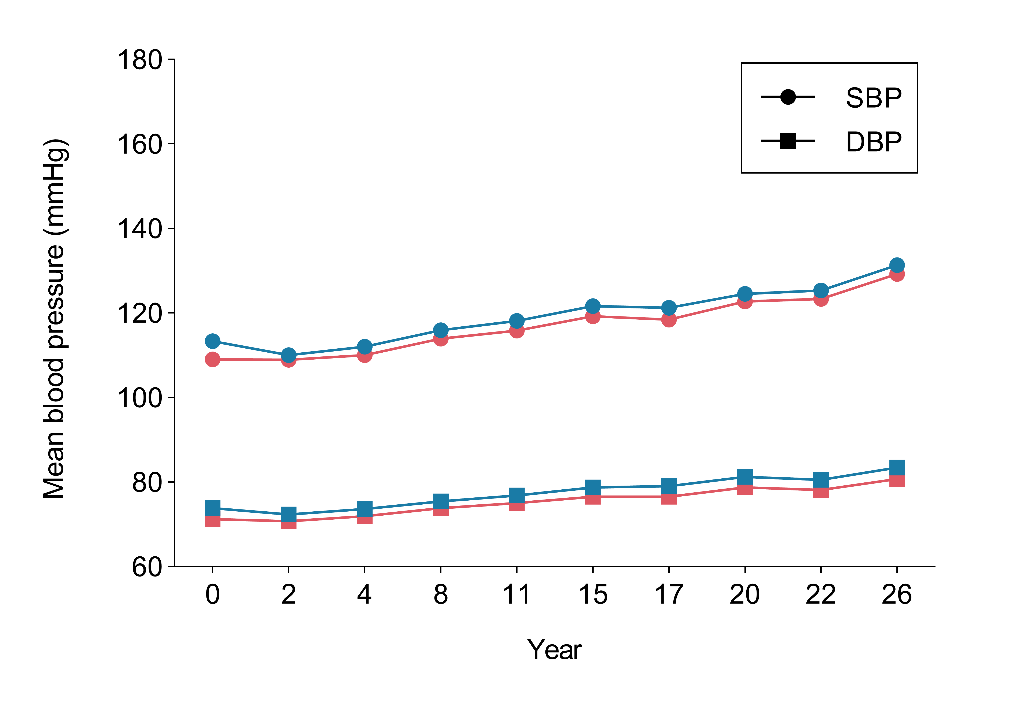


The blue lines represent men, and the red lines represent women.

Abbreviations: SBP, systolic blood pressure; DBP, diastolic blood pressure.
